# Dengue in Thailand and Cambodia: An Assessment of the Degree of Underrecognized Disease Burden Based on Reported Cases

**DOI:** 10.1371/journal.pntd.0000996

**Published:** 2011-03-29

**Authors:** Ole Wichmann, In-Kyu Yoon, Sirenda Vong, Kriengsak Limkittikul, Robert V. Gibbons, Mammen P. Mammen, Sowath Ly, Philippe Buchy, Chukiat Sirivichayakul, Rome Buathong, Rekol Huy, G. William Letson, Arunee Sabchareon

**Affiliations:** 1 Pediatric Dengue Vaccine Initiative, International Vaccine Institute (IVI), Seoul, Korea; 2 Armed Forces Research Institute of Medical Sciences (AFRIMS), Bangkok, Thailand; 3 Institut Pasteur in Cambodia, Phnom Penh, Cambodia; 4 Faculty of Tropical Medicine, Mahidol University, Bangkok, Thailand; 5 Bureau of Epidemiology, Ministry of Public Health, Nonthaburi, Thailand; 6 National Dengue Control Program, Ministry of Health, Phnom Penh, Cambodia; Tropical Medicine Institute Pedro Kourí (IPK), Cuba

## Abstract

**Background:**

Disease incidence data are needed to guide decision-making for public health interventions. Although dengue is a reportable disease in Thailand and Cambodia, the degree that reported incidence underrecognizes true disease burden is unknown. We utilized dengue incidence calculated from laboratory-confirmed outpatient and inpatient cases in prospective cohort studies to estimate the magnitude of dengue underrecognition and to establish more accurate disease burden estimates for these countries.

**Methods and Findings:**

Cohort studies were conducted among children aged <15 years by members of a dengue field site consortium over at least 2 dengue seasons. Age-group specific multiplication factors (MFs) were computed by comparing data from three cohort studies to national surveillance data in the same province and year. In Thailand, 14,627 person-years of prospective cohort data were obtained in two provinces and 14,493 person-years from one province in Cambodia. Average annual incidence of laboratory-confirmed dengue was 23/1,000 and 25/1,000 in Thailand, and 41/1,000 in Cambodia. Calculated MFs in these provinces varied by age-group and year (range 0.4–29). Average age-group specific MFs were then applied to country-level reporting data and indicated that in Thailand a median 229,886 (range 210,612–331,236) dengue cases occurred annually during 2003–2007 and a median 111,178 (range 80,452–357,135) cases occurred in Cambodia in children <15 years of age. Average underrecognition of total and inpatient dengue cases was 8.7 and 2.6-fold in Thailand, and 9.1 and 1.4-fold in Cambodia, respectively. During the high-incidence year 2007, >95,000 children in Thailand and >58,000 children in Cambodia were estimated to be hospitalized due to dengue.

**Conclusion:**

Calculating MFs by comparing prospective cohort study data to locally-reported national surveillance data is one approach to more accurately assess disease burden. These data indicate that although dengue is regularly reported in many countries, national surveillance data significantly underrecognize the true burden of disease.

## Author Summary

Dengue is a major public health problem especially in tropical and subtropical countries of Asia and Latin-America. An effective dengue vaccine is not yet available, but several vaccine candidates are currently being evaluated in clinical trials. Accurate country-level incidence data are crucial to assess the cost-effectiveness of such vaccines and will assist policy-makers in making vaccine introduction decisions. Existing national surveillance systems are often passive and are designed to monitor trends and to detect disease outbreaks. Our analyses of data from prospectively followed cohorts with laboratory confirmation of dengue cases show that, in Thailand and Cambodia, dengue incidence is underrecognized by more than 8-fold. The magnitude of the outpatient burden caused by dengue is not assessed or reflected by the national surveillance data. We estimate that a median of more than 340,000 symptomatic dengue virus infections occurred annually in children less than 15 years of age in Thailand in Cambodia between 2003 and 2007.

## Introduction

Dengue is a mosquito-borne viral disease that is increasing in incidence and economic importance [1]. It is endemic in most tropical areas of Asia, the Americas, and some parts of Sub-Saharan Africa [1]. Most dengue virus (DENV) infections occur either asymptomatically or present clinically as undifferentiated fever, followed, in increasing rarity, by classic dengue fever (DF) and dengue hemorrhagic fever (DHF) [2].

Evidence of the magnitude and trends of diseases should contribute to decision-making at the global and national levels, especially in the context of increasing health care costs and increasing availability of effective interventions [3]. For dengue, it is estimated that about 50–100 million individuals are infected annually worldwide with up to 500,000 people being admitted to hospital [4]. However, it is generally believed that these numbers still represent a large underestimate of the actual disease burden. In most countries, reporting of dengue is based solely on clinical criteria. The variable clinical picture of dengue, and the diagnostic confusion with other similarly presenting febrile diseases, complicates dengue disease surveillance [5]. In addition, in some countries only hospitalized dengue cases are reported to national surveillance systems. This is usually sufficient, since the primary objective of these systems is to detect epidemics, guide immediate actions, and monitor trends [5].

Due to the underrecognition of cases, data drawn from national or regional surveillance and reporting systems are usually not sufficient to be used for disease or economic burden estimates. To overcome this problem, authors of several published dengue studies multiplied the number of reported cases by a set factor to obtain an estimate of actual cases [6]–[8]. Usually these multiplication factors (MFs) have been based on data derived from a single study, often performed in a different country, or based on “expert opinion” [6], [7]. For example, the authors of a study that assessed the disability adjusted life years lost to dengue in Brazil used a range of MFs (from 0.3 through 10) with the upper limit estimate derived from a cohort study in Thailand, given the uncertainties regarding the magnitude of disease underrecognition in their country [8]. Attempts to systematically assess such MFs, for example through capture-recapture studies, have only rarely been undertaken and usually have not taken outpatients into account [9]. But a recent study from Thailand clearly demonstrated that non-hospitalized patients with dengue illness represent a substantial proportion of the overall disease burden [10].

Over the past 30 years dengue has been a major public health problem in Thailand and Cambodia, where children below 15 years of age are especially affected [11], [12]. In both countries DF and DHF cases are notifiable. In Thailand, dengue case surveillance is mainly based on clinical and general laboratory criteria, and there is an emphasis on hospitalized cases in spite of encouragement for outpatient reports [12]. In Cambodia, only hospitalized children <15 years of age are reportable to the Ministry of Health (MoH).

The Pediatric Dengue Vaccine Initiative (PDVI), a product development partnership of the International Vaccine Institute (IVI) in Seoul, Korea, whose goal is to accelerate the development, evaluation and introduction of dengue vaccines, has established a field site consortium, which includes three member field sites from Thailand and Cambodia, each having conducted at least two years of active dengue surveillance within defined cohorts as of 2008. The objective of this study was to utilize laboratory-confirmed incidence of symptomatic DENV infection both in inpatients and outpatients identified in prospective cohort studies at these three field sites to estimate the magnitude of dengue underrecognition in Thailand and Cambodia. Accurate country-specific incidence data are crucial for the assessment of the economic impact of dengue and the cost-effectiveness of a potential dengue vaccine in the future.

## Methods

To estimate the true number of dengue cases in Thailand and Cambodia, we compared field site data with reported data on the provincial level in order to establish age-group specific MFs. Prospective cohort data were used from three provinces: Kamphaeng Phet and Ratchaburi in Thailand, and Kampong Cham in Cambodia.

### The two cohort studies in Thailand

**Figure 1:**
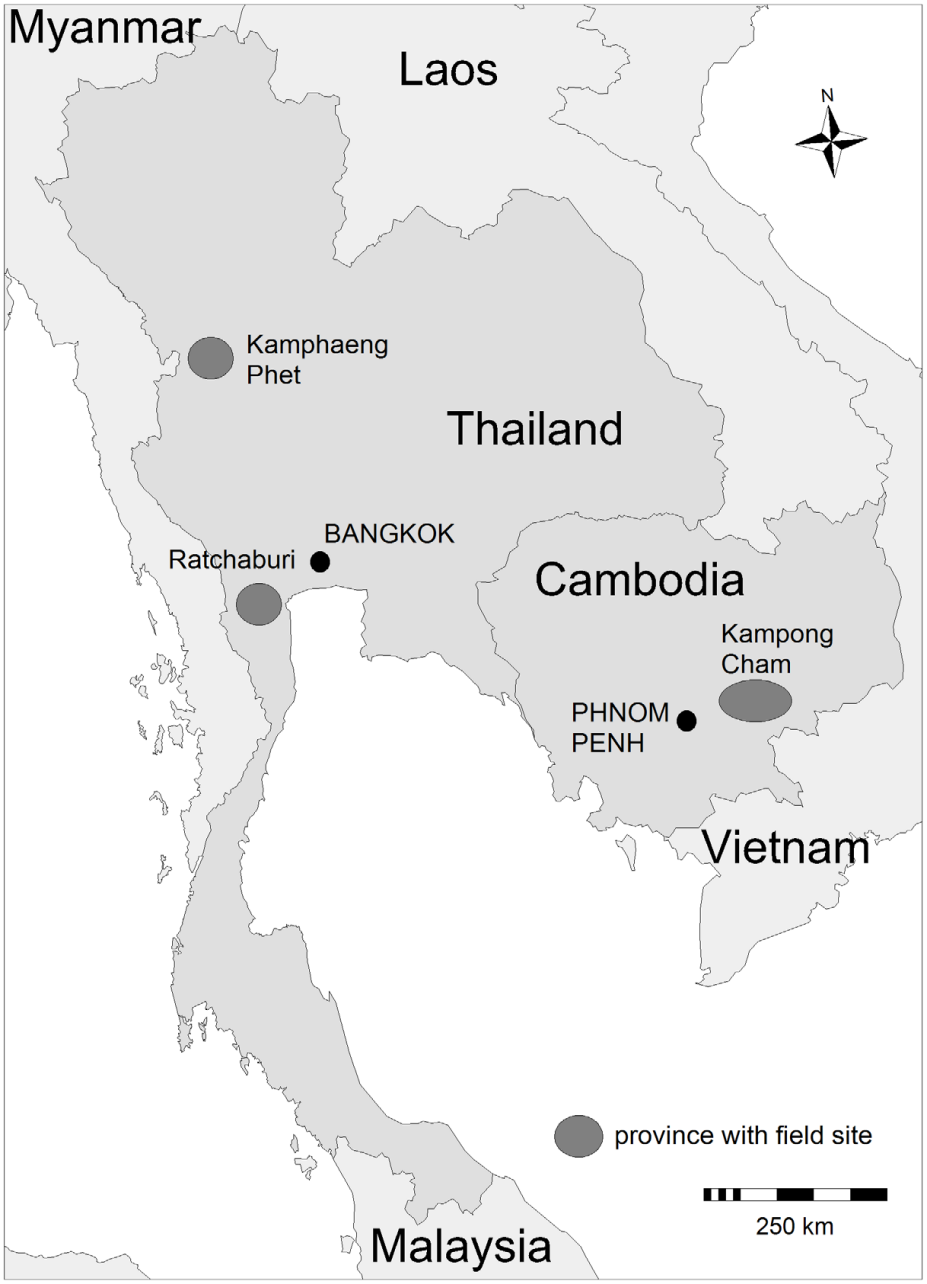
Geographic location of provinces with dengue field sites in Thailand and Cambodia. The field sites were members of a dengue field site consortium and provided prospective cohort data to be compared with the dengue reporting data in the same province.

Prospective data from two cohort studies in Thailand were included in the analysis. Demographics for these two sites are based on population census data as of 2000 [13]. One study has been conducted since 1998 in Muang district of Kamphaeng Phet Province, located 350 km northwest of Bangkok (Figure 1). By Thai standards, the study area is relatively sparsely populated with 206,271 residents in 56,874 households in an area encompassing 1,384 km^2^ (population density: 149/km^2^). Approximately 30% of the total provincial population lives in Muang district (province population density: 78/km^2^). Further details of this study sites and the cohort study designs have been published previously [14]. The other cohort study has been carried out since 2005 in Muang district of Ratchaburi Province, which is located 100 km west of Bangkok at the Thai-Myanmar border (Figure 1). A total 183,528 residents live in 47,608 households in the urban Muang district (area of 418 km^2^, population density: 439/km^2^). Approximately 23% of the total provincial population lives in Muang district (province population density: 161/km^2^). To compare the cohort data with provincial reporting data, we included in the analysis data gathered within the Kamphaeng Phet cohort between 2004 and 2007 (in 2003 no dengue surveillance was conducted within this cohort) and within the Ratchaburi cohort between 2006 and 2007.

In Kamphaeng Phet, approximately 2,000 children from kindergarten to grade five (age-range: 4 to 13 years of age) at 11 local primary schools were recruited into a dynamic cohort in January 2004. New participants were enrolled from the kindergarten class in January of each year to replace subjects who graduated from the sixth grade. In Ratchaburi, the cohort was fully established in February 2006 including 3,026 students aged 3–14 years attending 7 local schools. Children who left the cohort in 2006 were replaced in February of the following year with students in the same age-range. For both cohort studies, sample size calculations based on expected dengue incidences in the target population.

In both cohorts, acute dengue illness was identified on the basis of absence from school or a visit to the school nurse or the involved health centers (Kamphaeng Phet) and regional hospital (Ratchaburi). While active case surveillance of study participants for acute illness occurred in Ratchburi throughout the whole year, surveillance in Kamphaeng Phet was limited to the dengue season from June to November each year.

In both studies, acute-illness and convalescent (i.e. 10–21 days later) blood samples were obtained from all students with a history of fever within the previous 7 days or an oral temperature of ≥38°C after obtaining signed parental consent. In Ratchaburi, however, in the first year blood was only collected from students with clinically suspected dengue (i.e. fever for >2 days and no localizing signs, or any child fulfilling the WHO dengue clinical case definition [15]). DENV-infection was confirmed in the Kamphaeng Phet study by using the AFRIMS in-house dengue IgM/IgG enzyme immunoassay (EIA) in acute and convalescent sera, or reverse-transcriptase polymerase chain reaction (RT-PCR)/nested PCR in the acute serum sample modified from the Lanciotti procedure, or virus isolation in the acute serum sample as described previously [14]. In Ratchaburi, infections were confirmed by the detection of dengue-specific IgM/IgG antibody using in-house EIAs or of DENV using virus isolation or RT-PCR with Lianciotti primers [16]. Both study protocols were reviewed and approved by the ethics committee of the Thai Ministry of Public Health (MoPH). In addition, the Ratchaburi protocol was approved by the Institutional Review Board of IVI, and the Kamphaeng Phet protocol was approved by the ethical review committees of the U.S. Army Surgeon General, University of California at Davis, University of Massachusetts Medical School, and San Diego State University.

### The cohort study in Kampong Cham, Cambodia

A prospective community-based active surveillance study was performed in 16 rural villages of Kampong Cham Province, Cambodia, between May to November 2006. Included were approximately 9,000 children aged 0–15 years. Additional details of this cohort study have been published previously [17]. In 2007, the study was carried out between June and December and was expanded to around 10,000 children aged 0–19 years from 20 rural and 5 urban villages. The sample size calculation based on expected dengue incidence in the target population and took a cluster design effect of 2 into account due to the expanded catchment area and the inclusion of urban and rural areas. Villages under surveillance were located in 3 districts of Kampong Cham Province (total population 1.7 million), which is located approximately 170 km northeast of Phnom Penh (Figure 1). For the study, a convenience sample of villages from two rural districts located within an approximately 60 km radius of the capital town and the capital town's urban areas was selected. Selected urban areas' population density was estimated at ∼1,900/km^2^ versus ∼450/km^2^ within rural villages (with the geographic area limited to the villages and not –in contrast to the above presented data from Thailand– including the geographic area of the entire administrative district, making the population density estimates higher for Cambodian villages). After obtaining consent for participation from village chiefs and their elders' council, a field team visited on a weekly basis all households under fever surveillance and took temperature of any sick child enrolled in the study. In 2007, digital thermometers and temperature logbooks were additionally provided to participating households to record any suspected fever occurring between two visits. From children with fever (i.e. ≥38°C, acute or in the previous 7 days) for ≥2 days (in 2006) or 1 day (in 2007), acute and convalescent phase serum samples were collected by an investigation team after obtaining signed parental consent. All acute and convalescent serum specimens were tested for anti-DENV IgM using an in-house capture EIA (MAC-ELISA). RT-PCR testing was performed only on acute phase specimens that were anti-DENV IgM-negative and where the convalescent sample was IgM-positive in order to conserve reagents, followed by cell culture for isolation when appropriate. RT-PCR was performed using a modified Lanciotti procedure as described earlier [18], [19]. Dengue viruses were isolated after inoculation of the sera into C6/36 (Aedes albopictus) and VERO E6 cells cultures followed by virus serotype identification by direct fluorescent antibody assay using monoclonal antibodies. Since both DENV and Japanese Encephalitis virus (JEV) are co-circulating in Cambodia and because a serological cross-reactivity between these two flaviviruses can be observed, all specimens were systematically tested for anti-DENV and anti-JEV IgM by an in-house MAC-ELISA as described previously [19]. Symptomatic DENV infection was defined as documented fever and the detection of either anti-DENV IgM antibodies in the second serum sample or the detection of DENV in acute serum by RT-PCR or virus isolation. The study protocol was reviewed and approved by the Ethical Committee of the MoH Cambodia and the Institutional Review Board of IVI.

### National surveillance data from Thailand and Cambodia

National and provincial level dengue reporting data were extracted from the national surveillance data base of the MoPH Thailand and the MoH Cambodia. National data were stratified by type of management (inpatient vs. outpatient), by year, and by age-group (0–4, 5–9, and 10–14 years). Provincial level data were stratified by type of management, by year (Kamphaeng Phet 2004–07, Ratchaburi 2006–07, Kampong Cham 2006–07), and by age-group (Kamphaeng Phet 5–9 and 10–12; Ratchaburi 0–4, 5–9, 10–14; Kampong Cham 0–4, 5–9, 10–14 years). Total population data (national and provincial level) were provided by both ministries stratified by age-group and year.

In Cambodia, the case definitions for reporting of DF and DHF are adapted from the WHO clinical case definition, but only hospitalized cases and cases <15 years of age are reported [17]. The case definitions are based only on clinical and hematological criteria (according to WHO guideline these are “suspected” dengue cases) and do not require laboratory confirmation. For DF, the presence of fever with 2 or more of the following signs is required: Red face or conjunctival injection, headache, retro-orbital pain, painful muscles or joints, rash, and hemorrhagic signs. Leucopenia may be present. For DHF, besides the above listed DF-signs and hepatomegaly or abdominal pain the following hematological findings are required: Increase in hematocrit ≥20% and drop in platelets below 100,000/mm^3^.

In Thailand, the adapted WHO clinical case definition is used to report both hospitalized and non-hospitalized patients of all ages. Clinical case definitions are available for DF and DHF (as described for Cambodia) and are supported by hematological criteria such as leucopenia for DF, or thrombocytopenia and increase in hematocrit >10–20% from baseline for DHF. Specific diagnostic tests (e.g. serological tests or RT-PCR) may be ordered on an individual basis by clinicians and depend on the capacity of the hospital. Subsequently, dengue cases can be classified as suspected (i.e. only based on clinical and hematological criteria), probable (clinical criteria plus supportive serology from a single blood specimen), and confirmed cases (confirmed by laboratory criteria). All three case classifications are applied for both DF and DHF and are used nationwide by all governmental hospitals and some private clinics and hospitals. A suspected dengue case will be removed from the surveillance data set, if appropriate laboratory testing for dengue reveals negative results.

### Data analysis

#### Calculation of multiplication factors

Incidence data from the field sites were stratified in the same manner as the provincial reporting data by type of management, year, and age-group. Two types of multiplication factors were computed. Multiplication factor 1 (MF1) accounts for underrecognition of inpatient dengue cases. Age-group specific MF1s were calculated by dividing the incidence of laboratory-confirmed inpatient dengue cases in the cohort by the reported incidence of dengue inpatient cases in the same province and year. Multiplication factor 2 (MF2) gives an estimate of the true number of outpatient dengue cases. Age-group specific MF2s were calculated by dividing the number of laboratory-confirmed dengue outpatients by dengue inpatients in the cohort by year. In Ratchaburi, blood samples were obtained in 2006 only from clinically-suspected dengue cases and these patients were routinely hospitalized at the Provincial Hospital. We therefore only calculated MF2 in Ratchaburi for 2007 when all febrile cases were tested for dengue.

#### Estimates for the true number of symptomatic dengue cases nationally

**Figure 2:**
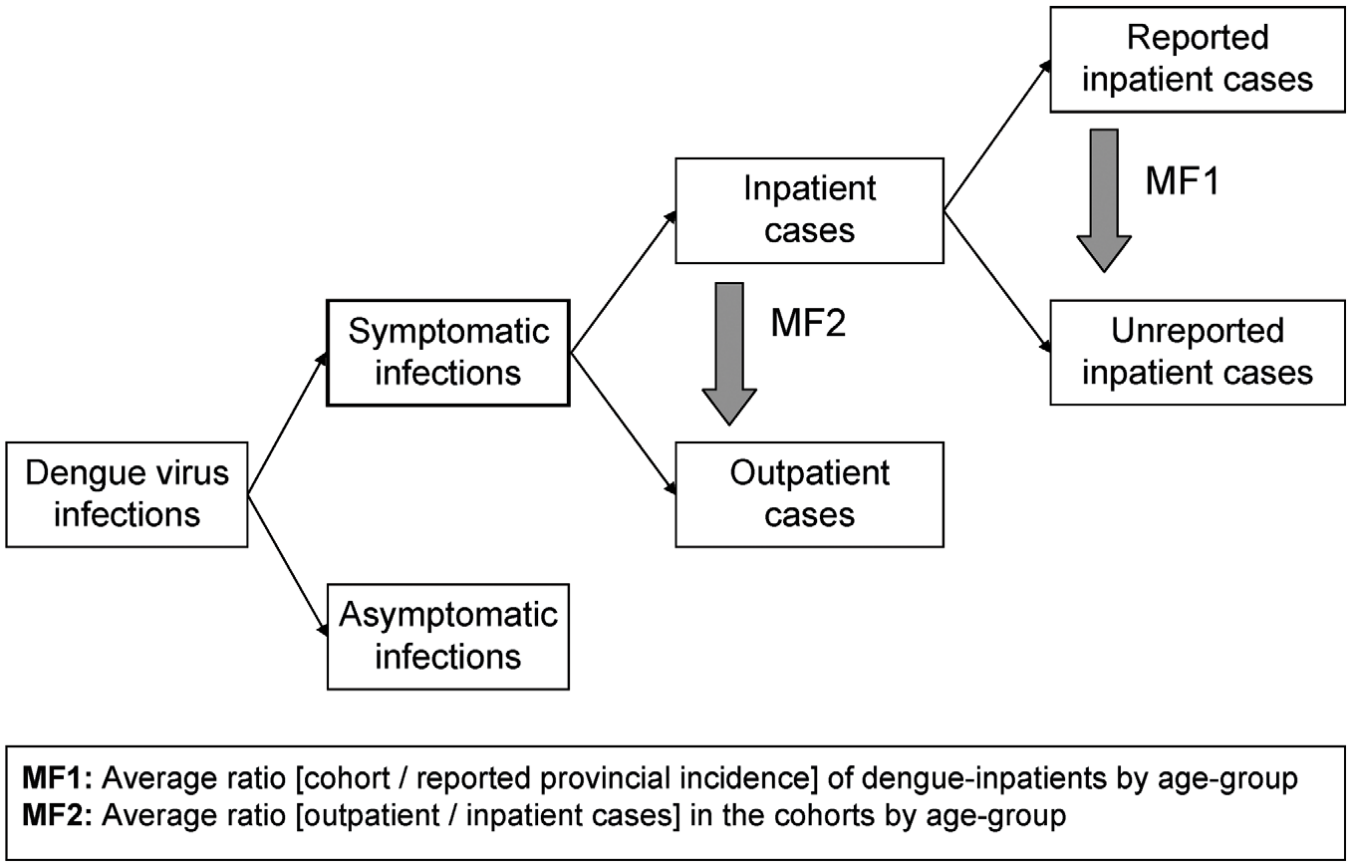
Methodology used to establish better disease burden estimates of symptomatic dengue virus infections. The estimates are based on numbers of nationally reported inpatient dengue cases and average multiplication factors (MF), which were generated by comparing provincial reporting data with data from prospective cohort studies in the same province.

To estimate the true number of dengue inpatients in the entire country, the reported national number of dengue inpatients was multiplied by the average MF1 for each age-group (0–4, 5–9, and 10–14 years) (Figure 2). For Kamphaeng Phet, the age-group 10–12 MF1 was assumed to be similar to an age-group 10–14 MF1. For Ratchaburi, the age-group 3–4 multiplication factors were assumed to be similar to age-group 0–4 multiplication factors. To estimate the true number of symptomatic outpatient dengue cases in the country, the estimated true number of dengue inpatients was multiplied with age-group specific average MF2 (Figure 2). Calculations were performed for each year 2003–07 with the same average age-group specific MF1 and MF2.

## Results

### Thailand

**Figure 3:**
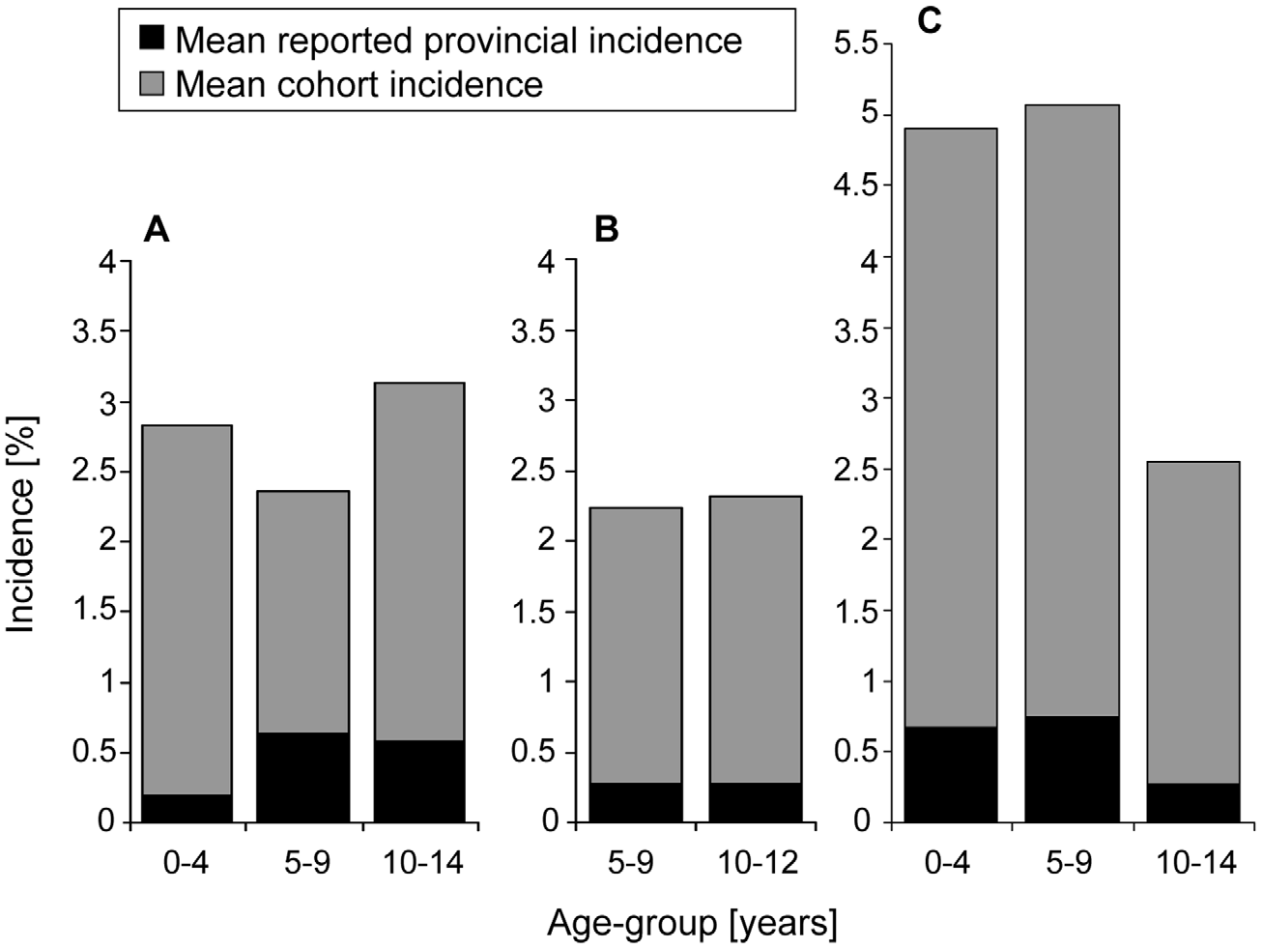
Dengue age-group specific incidence data in Thailand and Cambodia. Differences are shown between mean reported provincial incidence and mean cohort incidence of symptomatic dengue virus infections (inpatients and outpatients) by age-group under surveillance in three field sites: A) Ratchaburi, Thailand, 2006–07; B) Kamphaeng Phet, Thailand, 2004–07; C) Kampong Cham, Cambodia, 2006–07.

In Thailand, a cumulative total of 14,627 student-seasons (Kamphaeng Phet) or student-years (Ratchaburi) were studied in the two cohorts: 8,246 in Kamphaeng Pet 2004–07 and 6,381 in Ratchaburi 2006–07 (Table 1). The incidence of laboratory-confirmed symptomatic DENV infection (both inpatients and outpatients) ranged in both field sites between 13 and 33/1,000 with an average of 23/1,000 in Kamphaeng Phet and 25/1,000 in Ratchaburi. The average age-group specific incidence per cohort study is shown in Figure 3. The proportion of cohort subjects which were lost to follow-up, was in Kamphaeng Phet 2% to 2.5% (over the 6 month seasonal surveillance period) and in Ratchaburi approximately 11% (over the entire year in 2006).

**Table 1:** Age-group specific dengue incidence in three cohort studies and reported provincial incidence by year.

Province, country	Year	Age-group	Cohort subjects (n)	Total incidence in the cohort (per 1,000)	Cohort outpatient incidence (per 1,000)	Cohort inpatient incidence (per 1,000)	Reported province inpatient incidence (per 1,000)	MF1 (ratio cohort inpatient incidence: reported province inpatient incidence)	MF2 (ratio outpatients: inpatients in the cohort)
Kamphaeng	2004	5–9	1,383	19.5	15.9	3.6	2.0	1.8	4.4
Phet,		10–12	87	8.7	7.3	1.5	2.0	0.7	5.0
Thailand	2005	5–9	1,317	7.6	6.8	0.8	2.4	0.3	9.0
		10–12	755	22.5	17.2	5.3	1.3	4.0	3.3
	2006	5–9	1,254	43.1	34.3	8.8	3.4	2.6	3.9
		10–12	820	43.9	29.3	14.6	2.4	6.0	2.0
	2007	5–9	1,165	18.9	15.5	3.4	2.7	1.3	4.5
		10–12	865	17.3	15.0	2.3	4.3	0.5	6.5
	**Total**	**5**–**12**	**8,246**	**22.7**	**17.8**	**4.9**	**2.6**	**1.9**	**3.7**
Ratchaburi,	2006	0–4	265	3.8	0.0	3.8	1.5	2.5	NA*
Thailand		5–9	2,407	17.9	4.6	13.3	6.7	2.0	NA*
		10–14	355	19.7	2.8	16.9	4.5	3.8	NA*
	2007	0–4	335	44.8	23.9	20.9	2.0	10.3	1.1
		5–9	2,126	29.6	13.6	16.0	5.1	3.1	0.9
		10–14	893	37.0	21.3	15.7	6.4	2.4	1.4
	**Total**	**0–14**	**6,381**	**25.4**	**10.7**	**14.7**	**4.5**	**3.3**	**1.0**
Kampong	2006	0–4	1,685	18.4	9.5	8.9	4.1	2.2	1.1
Cham,		5–9	2,099	16.2	6.2	10.0	4.5	2.2	0.6
Cambodia		10–14	2,910	8.2	6.5	1.7	1.4	1.3	3.8
	2007	0–4	2,316	71.2	62.2	9.1	9.4	1.0	6.9
		5–9	2,834	76.2	66.7	9.5	10.3	0.9	7.0
		10–14	2,649	44.5	43.0	1.5	4.0	0.4	28.5
	**Total**	**0–14**	**14,493**	**40.6**	**34.2**	**6.4**	**4.6**	**1.4**	**5.3**

NA, not available.

*In 2006, only clinically dengue-suspected febrile cases that were hospitalized were tested for dengue virus infection in Ratchaburi.

In Kamphaeng Phet and Ratchaburi, the incidence of dengue leading to hospitalization was 4.9/1,000 and 14.7/1,000 and the average outpatient-inpatient ratios were 3.7∶1 and 1∶1, respectively. Age-group specific incidence cohort data by year are summarized in Table 1. The average reported provincial incidence of dengue inpatient cases over the study periods was 2.6/1,000 in Kamphaeng Phet and 4.5/1,000 in Ratchaburi.

Applying average MFs to age-group specific nationally reported dengue cases 2003–07 showed an average of 8.7-fold (SD 0.13) underrecognition of total symptomatic dengue cases in Thailand each year. The underrecognition of inpatient dengue cases was estimated to be 2.6-fold (SD 0.02) during this time period. The calculations suggest that in 2003–07 a median of actually 229,886 (range 210,612–331,236) symptomatic DENV infections occurred each year in Thailand in the age-group <15 years with up to 95,527 hospitalizations in 2007.

Average age-group specific MFs and the single steps in the calculation exemplified by using median numbers of reported dengue cases in 2003–07 are shown in Table 2. Since these calculations were based on median numbers of reported dengue cases, the multiplication factors presented in the table (for total cases 8.37 and for inpatient cases 2.94) varied slightly from the more precise calculations presented above, which were conducted for each individual year 2003–07.

**Table 2:** Median reported dengue cases and application of age-group specific multiplication factors (MF), Thailand 2003–2007.

	(a)	(b)	(c)	(d)	(e)	(f)	(g)		
Age-group	Totalnationally reported cases	Total nationally reported inpatient	Average MF1	Estimated actual number of inpatients (b x c)	Average MF2	Estimated actual number of outpatients (d x e)	Estimated actual number of total cases (d+f)	Multiplication factor tota cases (g/a)	Multiplication factor inpatient cases (d/b)
0–4	3,120	2,699	6.40	17,274	1.14	19,692	36,966	11.85	6.40
5–9	10,257	9,859	2.03	20,014	3.15	63,043	83,057	8.10	2.03
10–14	14,309	10,044	2.95	29,630	2.77	82,075	111,704	7.81	2.95
**Total**	**27,686**	**22,602**		**66,917**		**164,810**	**231,727**	**8.37**	**2.94**

### Cambodia

In Cambodia, cohort data from 2006 and 2007 were included in the analysis and were derived from a cumulative 14,493 person-season under active surveillance. The proportion of children with refusals or loss to follow-up during the study period was 1.3% in 2006 and 1.7% in 2007. The incidence of laboratory-confirmed dengue in 2006 and 2007 among children <15 years of age was 13/1,000 and 64/1,000, respectively. Average age-group specific incidence is shown in Figure 3, and age-group specific cohort and reported provincial incidence of dengue leading to hospitalization is shown by year in Table 1. The outpatient-inpatient ratio was 1.2∶1 in 2006 and 8.6∶1 in 2007.

Applying average MFs to age-group specific nationally reported dengue cases in each individual year 2003–07 suggest an average 9.1-fold (SD 0.16) underestimation of total number and 1.4-fold (SD 0.02) underestimation of inpatient dengue cases in Cambodia in 2003–07. These estimates suggest that in 2003–07 a median of 111,178 (range 80,452–357,135) symptomatic dengue virus infections occurred in Cambodia each year with a maximum 58,118 hospitalizations in 2007. Average age-group specific MFs and their application to expand nationally reported data (exemplified by using the median number of dengue case 2003–07) are shown in Table 3. Since these calculations were based on median numbers of reported dengue cases, the multiplication factors presented in the table (for total cases 9.27 and for inpatient cases 1.4) varied slightly from the more precise calculations presented above, which were conducted for each individual year 2003–07.

**Table 3:** Median reported dengue cases and application of age-group specific multiplication factors (MF), Cambodia 2003–2007.

	(a)	(b)	(c)	(d)	(e)	(f)	(g)		
Age-group	Total nationally reported cases*	Total nationally reported inpatients	Average MF1	Estimated actual number of inpatients (b x c)	Average MF2	Estimated actual number of outpatients (d x e)	Estimated actual number of total cases (d+f)	Multiplication factor total cases (g/a)	Multiplication factor inpatient cases (d/b)
0–4	4,044	4,044	1.60	6,470	3.96	25,623	32,093	7.94	1.60
5–9	5,170	5,170	1.55	8,014	3.81	30,531	38,545	7.46	1.55
10–14	2,781	2,781	0.85	2,364	16.15	38,176	40,540	14.58	0.85
**Total**	**11,995**	**11,995**		**16,848**		**94,330**	**111,178**	**9.27**	**1.40**

*In Cambodia only hospitalized dengue cases are reported to the national surveillance system.

## Discussion

Best data on disease incidence are usually derived from prospective community-based cohort studies that actively follow cohort-participants and use laboratory testing to confirm the diagnosis. Comparing cohort with national surveillance data can give an estimate for the underrecognition of the disease in the country. However, variation in environmental and socioeconomic conditions can occur in a country causing local differences in disease transmission and incidence [14]. Therefore, we established average age-group specific MFs by comparing cohort data with reporting data on the provincial level. By doing this, we estimated the magnitude of underrecognition of disease incidence in the national reporting system of the province, which should not be severely influenced by the exact level of disease incidence. However, factors potentially having an impact on the degree of underrecognition include socio-economic characteristics of the population and access to health care, which might differ between urban and rural areas in the country. In Thailand, the cohort studies included children from both rural and urban areas. Both studies were conducted in populations of the capital districts with probably better access to hospitals and higher socio-economic status than in more remote areas, but on average the population in these districts does have a lower socio-economic status than for example the population of the Bangkok metropolitan area (in 2000, around 77% of the employed population of Kamphaeng Phet and 43% of Ratchaburi province worked in the agricultural sector) [13]. In Cambodia, areas with rural and urban characteristics of one large province were included, which may be representative of rural Cambodia, where 84% of the total population lives, but not be representative of the entire country.

The major finding of our analysis was that dengue incidence was underrecognized by more than 8-times in Thailand and more than 9-times in Cambodia. We conclude that the national surveillance systems in Thailand and Cambodia were efficient in capturing inpatient dengue case with only 2.6-fold and 1.4-fold underdetection, respectively. However, the surveillance system in Thailand largely underrecognizes the burden of dengue outpatients and the system in Cambodia does not allow reporting of outpatients at all. According to our estimates (Table 2 and 3), in both countries symptomatic dengue outpatients account for more than 70% of the overall dengue disease burden. It is important to highlight that national reporting systems are usually designed to detect outbreaks and to monitor disease incidence trends, but do not attempt to capture all symptomatic dengue infections in the country for obvious reasons. Therefore, country-specific multiplication factors derived from studies like ours or from capture-recapture studies are useful for application to national reporting data to assess true disease burden and to be used for economic assessments.

Estimating the underreporting of dengue inpatients on the provincial level (MF1) and the ratio of outpatients to inpatients (MF2) showed considerable variation between years and age-groups both within and between field sites. Possible reasons for these variations include health structure differences (in Ratchaburi most cases presented directly at the Provincial Hospital where all clinically-suspected dengue cases were hospitalized; while in Kamphaeng Phet only more severe cases were referred from the public health centers to the hospital), differences in socio-economic characteristics of the populations, and annual differences in dengue activity (e.g. the large 2007 dengue-epidemic in Kampong Cham led to overwhelmed hospitals, thus increasing the proportion of non-hospitalized dengue cases but also leading to an overestimation of dengue among hospitalized febrile patients). However, these differences probably reflect reality and average age-group specific MFs were comparable between the three field sites. The inclusion of several field sites and field sites with different study areas (rural and urban) accounted for some local variation in economic characteristics of the study population and health care structure (which might also lead to differences in the proportion of suspected dengue cases being hospitalized) and is a major strength of our assessment. Still, the included study areas might not be representative for the entire country, but should represent large parts of each country.

For Kamphaeng Phet, the multiplication factor for age-group 10–12 was assumed to be similar to that of age-group 10–14. Since multiplication factors assess the degree of underrecognition of the disease, we assumed that there is not a relevant difference in health seeking behavior or in the hospitalization rate when comparing children 10–12 with children 13–14 years of age. However, there might be significant differences in health seeking behavior for the age-group 0–4 years vs. 5–9 years. Therefore we used for Thailand only data from the Ratchaburi cohort to assess multiplication factors for the age-group 0–4 years, and did not expand multiplication factors from children older than 4 years of age that were derived in the Kamphaeng Phet study to the 0–4 year age-group not studied at that site.

For several reasons we believe that the figures we present still underestimate the true dengue disease burden. First, in Kamphaeng Phet and Kampong Cham the cohort studies were only conducted during the dengue season. The full-year cohort study in Ratchaburi and the provincial reporting data demonstrated that there is still some dengue transmission outside the season (data not shown). On a national level, between 25% and 30% of annually reported dengue cases were notified in 2006 and 2007 outside the typical dengue season (typical season: June to November) [20]. Second, in Ratchaburi and Kampong Cham the inclusion criteria were changed in 2007 accounting also for dengue in patients with undifferentiated fever. Thus, mild but symptomatic dengue cases were probably missed in 2006. Third, it can be assumed that the presence of active surveillance studies in the provinces led to higher awareness in hospitals and thus better compliance with dengue case reporting to the national reporting system. Fourth, for the incidence calculation we used as a denominator the number of children under surveillance in the beginning of the year or season and did not account for drop-outs. And finally, only cases aged 0–14 years were included in the calculation since older age-groups were not studied in the field sites, and for Cambodia older age-groups are not reported in the national surveillance system. In Thailand, between 15,000 and 27,800 cases in individuals older than 14 years were annually reported between 2003 and 2007 to the national surveillance system (data not shown) indicating a high disease burden also in older age-groups. On the other hand, some overestimation might have occurred in Cambodia since only two years were studied with a large outbreak occurring in one. Despite these limitations and considerations we believe that with this present study we were able to roughly assess the magnitude of dengue underestimation in Thailand and Cambodia. Interestingly, our numbers were in the same range as the mainly “expert-opinion based” multiplication factors (10 for cases 0–15 years) used in a dengue economic study in Puerto Rico [7].

In this study we did not attempt to distinguish between DF and DHF-cases. By focusing on the hospitalization status we address the issue of disease burden rather than pathophysiology [21]. Especially for economic studies the distinction between hospitalized and non-hospitalized patients is more important because costs of hospitalized DF-cases are more similar to hospitalized DHF-cases [10].

In conclusion, the annual incidence of symptomatic DENV-infection in children <15 years was comparable between the three field sites and on average above 20/1,000. This highlights the high burden dengue poses to this age-group in Southeast Asia and shows a similar magnitude of the disease as earlier cohort studies in the region [10], [22]–[25]. It needs to be stressed that disease burden, age distribution of cases, or ratios between mild and severe dengue might be different in other dengue-endemic regions such as in the Americas. A recently performed comparison of dengue incidence in a pediatric cohort in Nicaragua with data reported to the National Epidemiological Surveillance program of the Nicaraguan Ministry of Health identified an average multiplication factor of 21.3 with an incidence of 3.4 to 17.6 cases per 1,000 children in the cohort [26]. For the Americas especially, but also in countries like Thailand, it would be ideal to extend dengue cohort studies into adulthood to better assess disease burden for the total population.

An effective dengue vaccine is not yet available, but several vaccine candidates are currently under development [27], [28]. Accurate country-level disease incidence data are therefore urgently needed to assess the true burden of dengue, to calculate its economic impact and the cost-effectiveness of a potential dengue vaccine, and to guide policymakers in making vaccine introduction decisions. Calculating MFs by comparing prospective laboratory-based cohort data to locally-gathered reporting data (in our study provincial-level data) is one approach to account for the underrecognition in national surveillance and reporting systems. Capture-recapture studies including outpatients would be useful to confirm these findings.

## Supporting Information

STROBE checklist (0.09 MB DOC)
